# Supplementary material for: The topology of synergy: Linking topological and information-theoretic approaches to higher-order interactions in complex systems
Source: PLoS Comput Biol. 2025 Nov 13;21(11):e1013649. doi: 10.1371/journal.pcbi.1013649 (PMC12643269; doi:10.1371/journal.pcbi.1013649)
Supplement: S1 File — (PDF) [file pcbi.1013649.s001.pdf]

Supplementary information for: The topology of synergy

Thomas F. Varley[1,2], Pedro A.M. Mediano[3,4], Alice Patania[1,5], and Josh Bongard[1,2]

[1]Vermont Complex Systems Institute, University of Vermont, Burlington, VT, USA
[2]Department of Computer Science, University of Vermont, Burlington, VT, USA
[3]Department of Computing, Imperial College London, London, UK
[4]Division of Psychology and Language Sciences, University College London, London, UK
[5]Department of Mathematics, University of Vermont, Burlington, VT, USA

September 19, 2025

SI 1 Topology and geometry of a truncated sphere

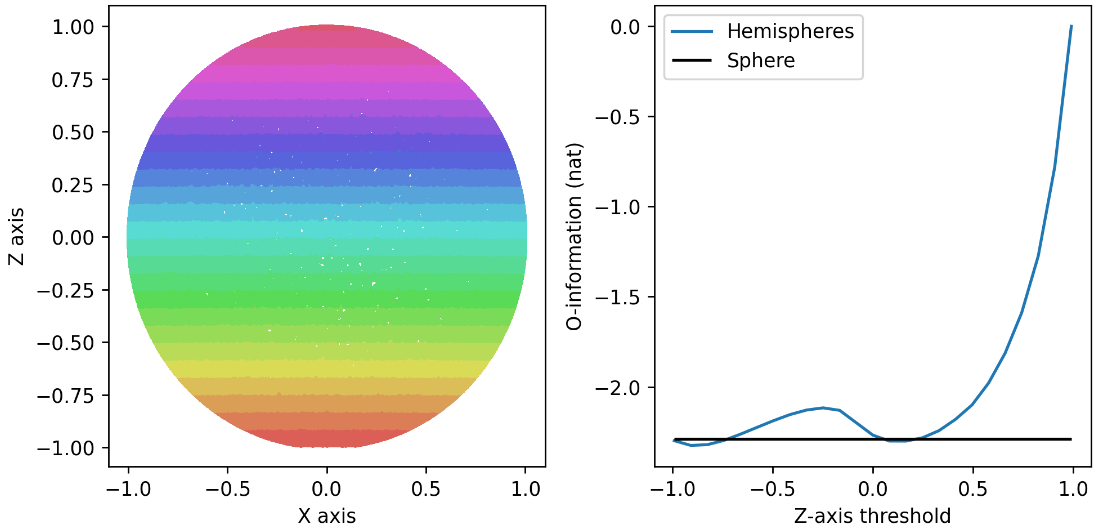

SI Figure 1:

There is a distinction to be made between the *topology* of a point cloud and the *geometry* of the point cloud. While our results strongly suggest that topology plays a significant role in the information-structure of a dataset, the geometry might also have an influence, as a point cloud can have "non-trivial" geometry, while technically having a "trivial" topology. The unit sphere

spans a range of [-1,1] on the z-axis. By successively removing all points below an increasingly larger thresholds on the [-1,1] interval, we can see how the O-information of a sphere changed as sections are removed (see SI Figure 1, left plot).

In SI Figure 1 (right plot), we can see that, as the geometry of the truncated sphere becomes increasingly plane-like (above the one-half threshold), the negative O-information rapidly drops to zero. However, when the threshold is below zero (indicating that more than 50% of the sphere is intact), the O-information remains strongly synergistic.

How can we interpret this? When less than 50% of the sphere has been removed and the z-threshold is below the equator, the topology of the truncated sphere technically becomes “trivial” in an absolute sense. However, since the K-nearest neighbors representation is an approximation, the appearance of a cavity can persist. We suspect that this is what is happening here; rather than there being an absolute difference between “non-trivial” and “trivial” topology, there is instead a spectrum that depends on the specific distribution of points and the particulars of the manifold-learning algorithm being used.

SI 2 Maximum persistence

The choice of average persistence lifetime of cavities is somewhat *ad hoc*, and there are other ways that the topological features of the point cloud can be characterized. To ensure the robustness of the results to specific analytical choices, we have replicated the correlations between information-theoretic and average persistence using the maximum persistence instead (the lifetime of the longest-lived cavity) instead.

The results of maximum persistence versus normalized O-information, dual total correlation, and dual total correlation are visualized in SI Figure 2. While the correlations are weaker, they remain highly significant for both redundancy-dominated and synergy-dominated triads, and all run in thei ame direction.

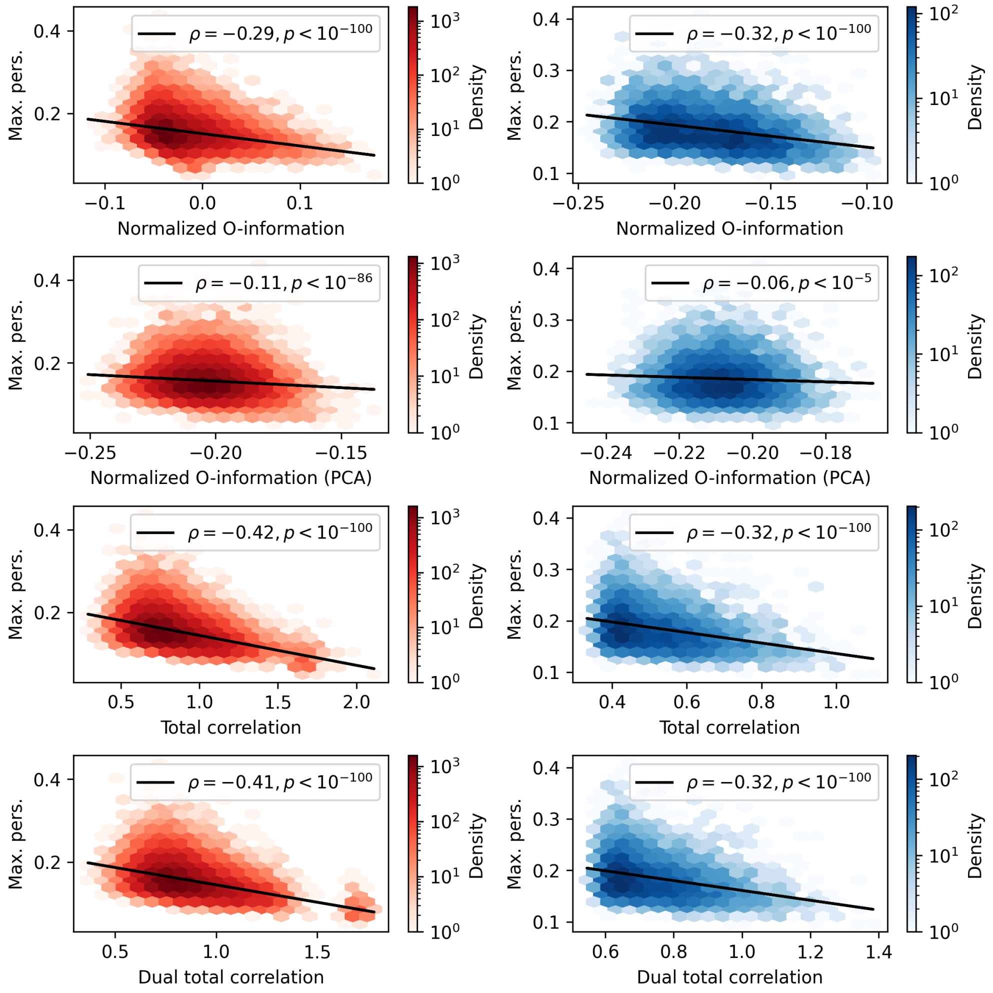

SI Figure 2: Hexbin plots using maximum persistence as opposed to average persistence. This should privilege the largest cavities.

SI 3 Replication of relationships with a second subject

The intensive computations required by both the information-theoretic and topological data analyses preclude replication with a large number of subjects. To ensure a base level of reproducibility, we repeated the analysis with four concatenated scans from another subject in the HCP dataset [?]. Upon comparing the correlations between the subjects, it is clear that the pattern of relationships liking informational and topological features are strongly preserved.

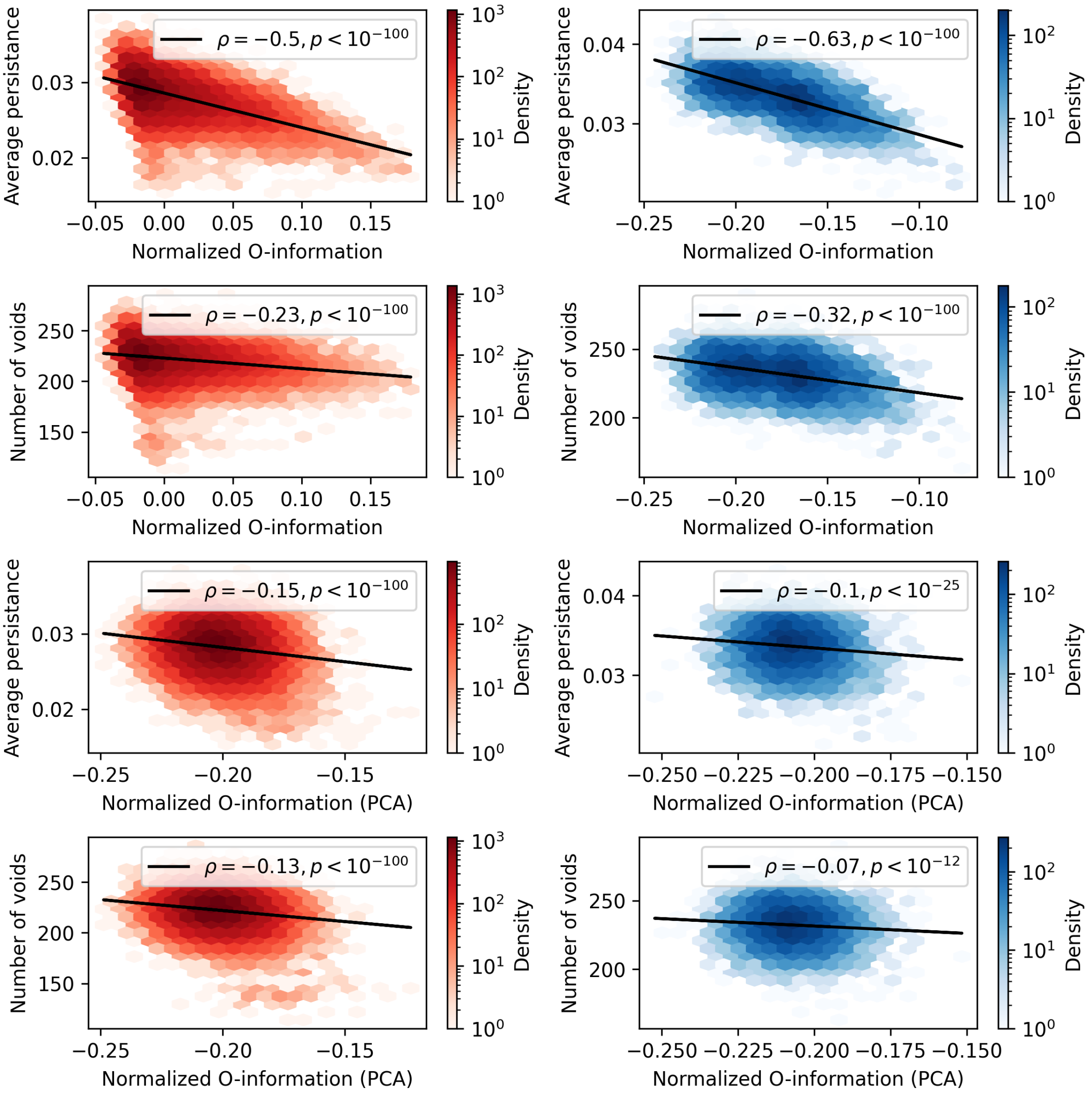

SI Figure 3: Compare to Fig. 7 in the main text.

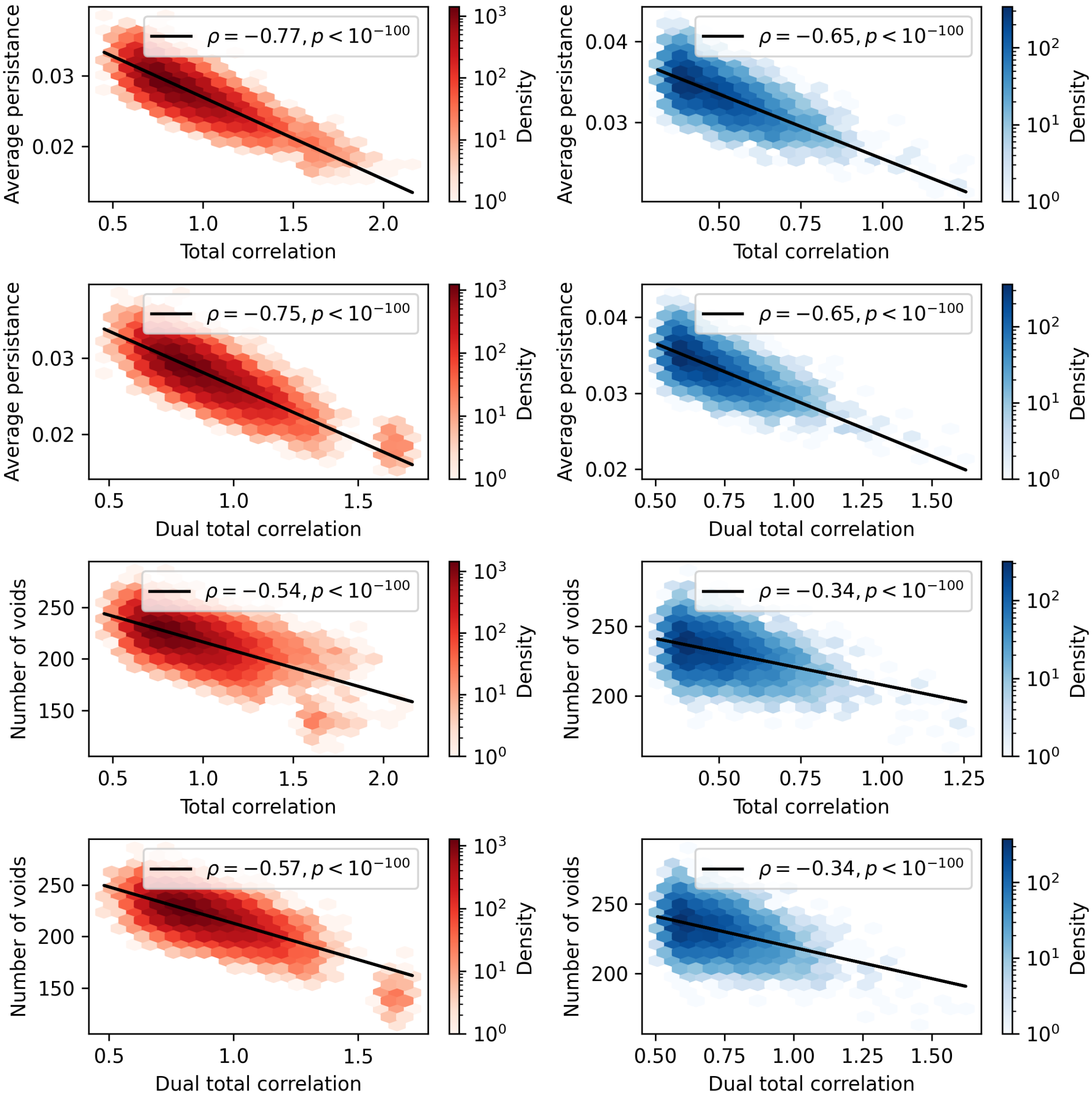

SI Figure 4: Compare to Fig. 6 in the main text.

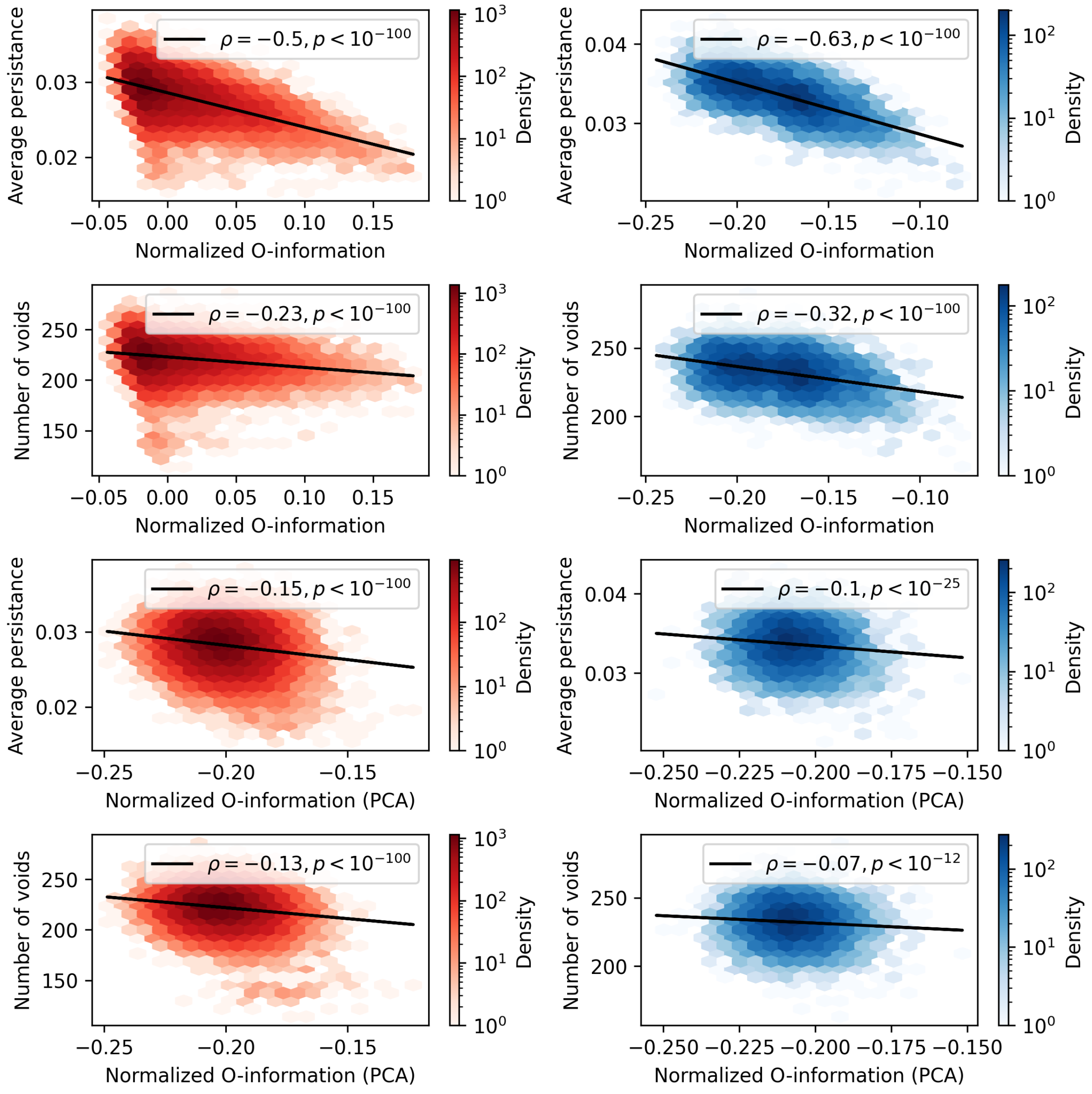

SI Figure 5: Compare to Fig. 7 in the main text.

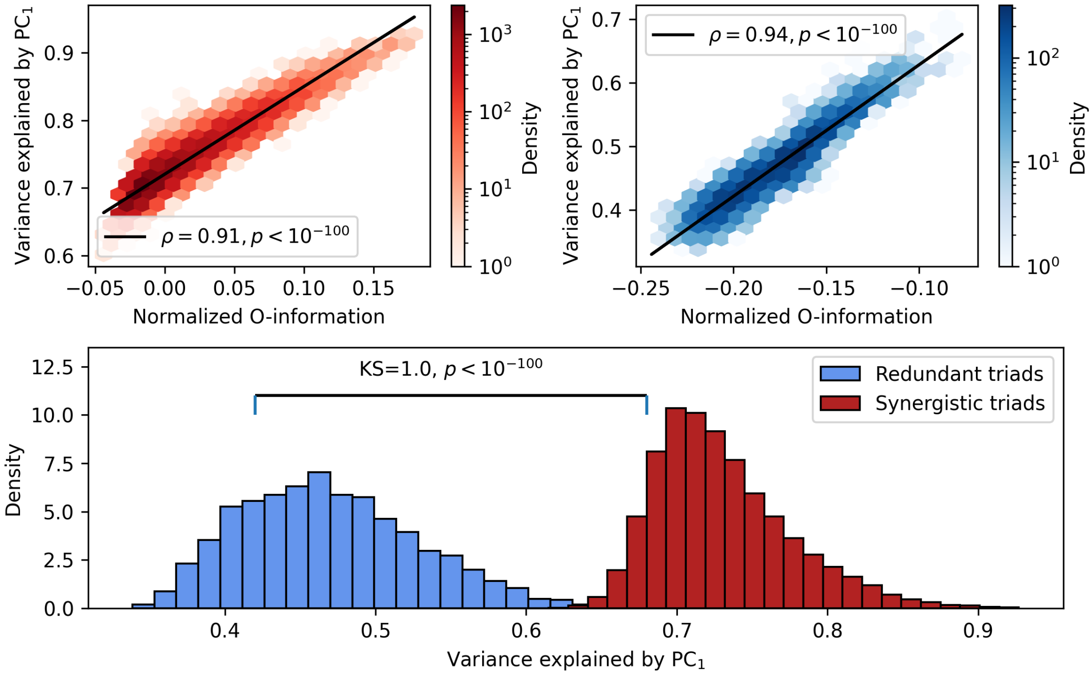

SI Figure 6: Compare to Fig. 8 in the main text.

SI 4 Network structure and normalized O-information

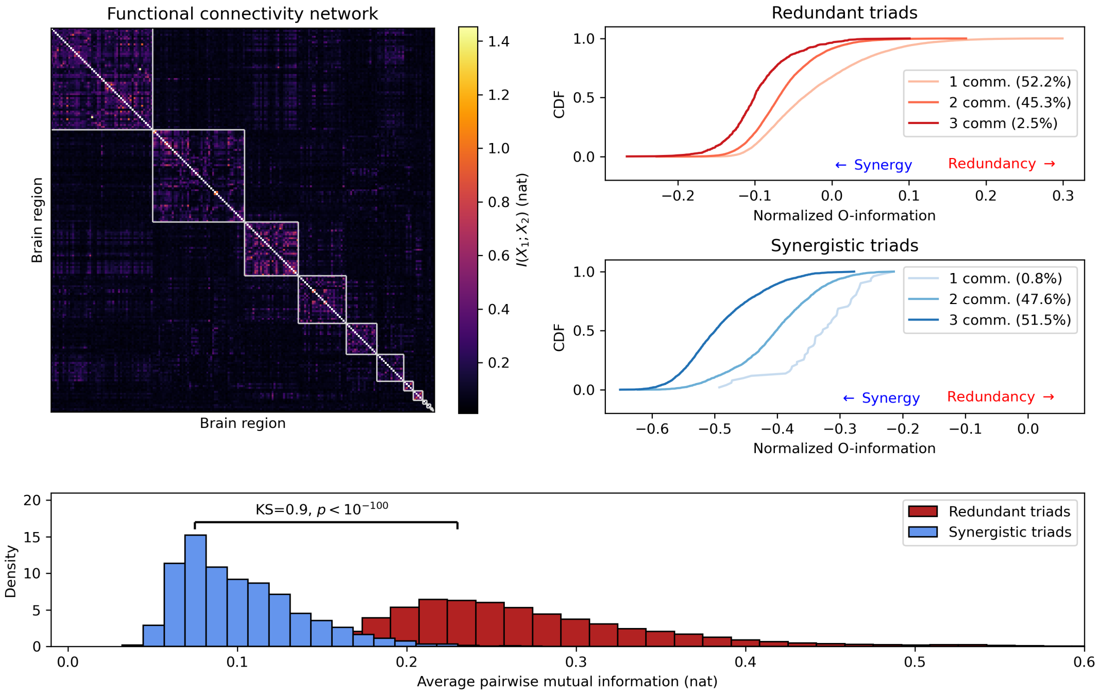

SI Figure 7: **The relationship between Kraskov O-information and bivariate functional connectivity structure. Top left:** The functional connectivity network constructed from an fMRI scan of a single person. Each edge gives the non-parametric, mutual information (estimated with the KSG algorithm [?]) between two brain regions. The matrix has been organized into functional clusters with higher within-module density and low between module density using multi-resolution consensus clustering [?]. **Top right:** We separated each of the triads in the redundancy- and synergy-dominated groups into triads that were contained within one functional community, spanned two functional communities, or had each element in three distinct communities. We then plotted cumulative distribution functions on the normalized O-information for each. We can see that, for redundant triads, those that spanned three communities had the weakest redundancy, while those that sat all within one community had the greatest redundancy. Similarly, for synergistic triads, those that spanned three communities had the greatest synergy, while those that all sat within one community (which were extremely rare, comprising les than 1% of synergistic triads) had the weakest synergy. **Bottom:** The distribution of pairwise mutual information (functional connectivity) for redundancy- and synergy-dominated triads was significantly different ($KS = 0.9$, $p < 10^{-100}$).
Collectively, these results replicate findings first reported by [?], using the non-parametric, continuous estimators as opposed to the previously used discrete estimator.

SI 5 PCA inverts the relationship between total correlation, dual total correlation, and topological features

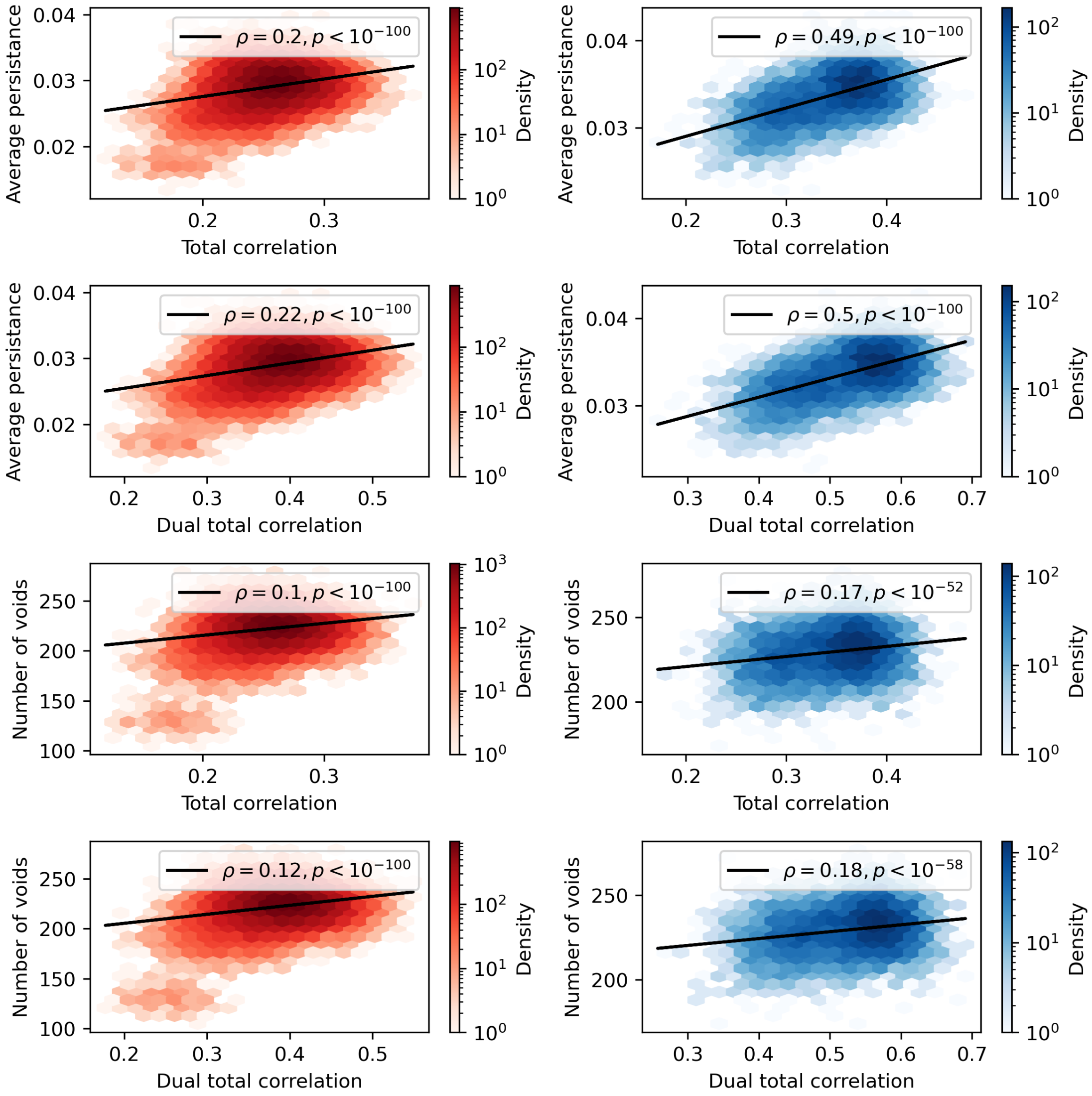

SI Figure 8: **Rotating point clouds inverts the relationship between TC, DTC, and higher-order topological features.** The same plot as shown in Fig. **??**, however the point-clouds have been rotated with PCA. Note that the direction of the relationship between multivariate information and topology reverses.

SI 6 Logical XOR gate analysis

$\mathbb{P}(\mathbf{X})$	X_1	X_2	X_3
1/4	0	0	0
1/4	0	1	1
1/4	1	0	1
1/4	1	1	0

Table 1: The lookup table for a stochastic logical XOR gate.

The logical XOR date is a classic example of an irreducibly higher-order structure in a multivariate system. For any pair of elements, the pairwise mutual information $I(X_i; X_j) = 0$ bit. However, the joint mutual information $I(\{X_i, X_j\}; X_k) = 1$ bit: there is information in the "whole" trivariate system that is not reducible to individual pairwise interactions. A "functional connectivity" network model of an XOR gate would be indistinguishable from a functional connectivity network of three independent processes. It is only when higher-order synergies are accounted for that the dependency structure becomes visible.
